# Comprehensive bioinformatic analysis reveals a fibroblast-related gene signature for the diagnosis of keloids

**DOI:** 10.1016/j.heliyon.2024.e35011

**Published:** 2024-07-22

**Authors:** Yue Qi, GuiE Ma

**Affiliations:** Plastic Surgery Hospital Chinese Academy of Medical Sciences, 33rd BaDaChu Street, Beijing, 100144, China

**Keywords:** Keloid, Fibroblast, Diagnostic model, Immune cell

## Abstract

**Aim:**

A keloid is a fibroproliferative cutaneous disorder secondary to skin injury, caused by an imbalance in fibroblast proliferation and apoptosis. However, the pathogenesis is not fully understood. In this study, candidate genes for keloid were identified and used to construct a diagnostic model.

**Methods:**

Three datasets related to keloids were downloaded from NCBI Gene Expression Omnibus. Fibroblast-related genes were screened, and fibroblast scores for the samples were determined. Then, a weighted gene co-expression network analysis (WGCNA) was used to identify modules and genes associated with keloids and the fibroblast score. Differentially expressed genes (DEGs) between keloid and control samples were identified and compared with fibroblast-related genes and genes in the modules. Overlapping genes were evaluated using functional enrichment analyses. Signature genes were further screened, and a diagnostic model was constructed. Finally, correlations between immune cell frequences and signature genes were analyzed.

**Results:**

In total, 124 fibroblast-related genes were obtained, and the fibroblast score was an effective indicator of the sample type. WGCNA revealed five modules that were significantly correlated with both the disease state and fibroblast scores, including 1760 genes. Additionally, 589 DEGs were identified, including 16 that overlapped with fibroblast-related genes and genes identified in the WGCNA. These genes were related to cell proliferation and apoptosis and were involved in FoxO, Rap1, p53, Ras, MAPK, and PI3K-Akt pathways. Finally, a six fibroblast-related gene signature (*CCNB1*, *EGFR*, *E2F8*, *BTG1*, *TP63*, and *IGF1*) was identified and used for diagnostic model construction. The proportions of regulatory T cells and macrophages were significantly higher in keloid tissues than in controls.

**Conclusion:**

The established model based on *CCNB1*, *EGFR*, *E2F8*, *BTG1*, *TP63*, and *IGF1* showed good performance and may be useful for keloid diagnosis.

## Highlights

• *CCNB1*, *EGFR*, *E2F8*, *BTG1*, *TP63*, and *IGF1* were associated with keloids.
• A diagnostic model based on the signature genes showed good performance.
• Proportions of regulatory T cells and macrophages were high in keloid tissues.

## 1 Introduction

A keloid is a fibroproliferative cutaneous disease following skin damage caused by a disruption in the balance between the proliferation as well as apoptosis of fibroblasts, resulting in excessive collagen deposition in the dermis and subcutaneous tissues [1]. Its clinical manifestations are scar hyperplasia and infiltrative growth outside the wound [2]. Keloid does not fade gradually and is often accompanied by itching as well as pain [2]. Importantly, uncontrolled keloids can contribute to functional impairment and a heavy burden on life [3]. Current treatments for keloids contain chemotherapy, surgery, radiotherapy, as well as stress therapy [4]. Although the treatment options can delay the progression of keloid, the recurrence rate is still high [5]. At present, the potential pathogenesis of keloid remains fully understood; therefore, in-depth investigations of the molecular mechanisms are necessary to promote the exploitation of novel treatment options and improve prognosis.

Multiple transcription factors, growth factors, as well as cytokines are reported to participate in wound healing [6]. Fibroblasts show differences in regulatory activity between normal wound healing and keloid tissues [7,8]. During injury, dermal fibroblasts are activated to become myofibroblasts which can expressα-SMA, and then propagate and migrate into the wound, thus depositing the complex extracellular matrix (ECM) components, as well as forming a signaling niche [9,10]. Once the integrity of the injury tissues is re-established, fibroblasts will become inactivated or begin to clear themselves [11]. On the contrary, if keloid fibroblasts always keep activated, and continuously secrete ECM, which can lead to excessive scar tissues [12]. Therefore, we can conclude that fibroblasts are the crucial effector cells in the formation of keloids; as well as targeting keloid fibroblasts may be a new therapy method for the treatment of keloids [13]. In addition to fibroblasts, genetic factors have pivotal functions in the occurrence of keloids [14]. Some genes related to fibrosis are associated with keloids [15,16]. For instance, the TGF-β/SMADS signaling pathway has the close relation with the keloids. TGF-β is found to cause fibrosis in fibroblasts of keloids, participate in the SMAD signaling pathway, and enhance the expression of a variety of collagen genes as well as their corresponding protein productions [17]. Additionally, a series of genome-wide association studies have been employed to identify numerous genomic susceptibility loci for the occurrence of keloids. For example, neuronal precursor cell-expressed developmentally downregulated 4 (*NEDD4*) has been identified to be a candidate gene; it enhances the proliferation as well as the invasion of fibroblasts; while activates the transcriptional activity of TGF-β/catenin [18,19]. However, genes related to fibroblasts in keloids have not been determined.

In the keloids, a chronic inflammatory state is common, in which multiple kinds of immune cells as well as cytokines are involved in the formation and development of keloids [20,21]. Excavating the alterations of keloid immune microenvironment can not only go deep into the pathogenesis of keloids, but also promote the treatment of keloid patients and improve the prognosis. As a result, this research screened a keloid fibroblast-related gene signature; as well as based on these genes, a diagnostic model was constructed for keloids. Furthermore, correlations between the identified signature genes and immune cell subsets were further explored. This study improves our understanding of the formation and progression of keloids and lays a foundation for diagnosis and treatment.

## 2 Data and methods

### 2.1 Expression profile data

On December 10, 2023, three datasets (GSE7890, GSE44270, as well as GSE212954) were downloaded from NCBI Gene Expression Omnibus (GEO) database. Among them, the GSE7890 dataset contained 19 relevant samples; 10 samples without drug treatment were selected, including 5 normal controls and 5 keloid regional tissue samples [22]. The detection platform for the GSE7890 dataset was the GPL570 Affymetrix Human Genome U133 Plus 2.0 Array. For the dataset of GSE44270, 12 samples were chosen, including 3 normal controls and 9 keloid region tissue samples, as well as its detection platform was the GPL6244 Affymetrix Human Gene 1.0 ST Array [23]. Additionally, the GSE212954 dataset contained 11 relevant samples, and 8 samples with clinical information were selected, including 4 normal controls and 4 keloid region tissue samples. The detection platform for the GSE212954 dataset was GPL20301Illumina HiSeq 4000. In total, 12 (5 + 3 + 4), and 18 (5 + 9 + 4) samples were incorporated in the control as well as keloid groups, respectively.

Because these datasets were derived from different batches of gene expression data, the sva package 3.38.0 in R 4.3.1 [24] was used to remove the batch effect on these three datasets; then, the combined expression data were obtained.

### 2.2 Evaluation of sample fibroblast scores

The gene set involved in fibrogenesis was downloaded from MSigDB in the Gene Set Enrichment Analysis (GSEA) database [25]. Then, the fibroblast scores for these samples were assessed through GSVA version 1.36.3 in R 4.3.1 [26]. Differences in the distribution of fibroblast scores between keloid as well as control samples were evaluated by the Kruskal–Wallis test. Additionally, the ability of fibroblast scores to identify sample types was analyzed using the receiver operator characteristic (ROC) curve method via pROC package version 1.12.1 in R 3.6.1 [27].

### 2.3 Weighted gene co-expression network analysis (WGCNA)

According to the all genes obtained in the combined dataset, WGCNA package version 1.61 [28] in R 4.3.1 was utilized to filtrate modules connected with the disease. The WGCNA algorithm was performed according to the steps of defining the adjacency function and module partition. The thresholds of module partitions were as follows: the genes in the module set ≥200, as well as cutHeight = 0.995. After that, the correlations among each module, fibroblast score, as well as disease state were calculated, and the modules with significant correlations with both the disease state and fibroblast score were retained.

### 2.4 Analysis of differentially expressed genes

In the combined expression profile dataset, the DEGs between keloid and control samples were filtrated using the limma package version 3.34.7 in R 4.3.1 [29], with the thresholds of false discovery rate (FDR) < 0.05 and |log_2_ fold change (FC)| > 0.5. Afterwards, the identified DEGs were compared to the genes in modules obtained from WGCNA and fibroblast-related genes from GSEA database to acquire overlapping genes. Following, the overlapping DEGs were utilized for Gene Ontology (GO), as well as Kyoto Encyclopedia of Genes and Genomes (KEGG) pathway enrichment analyses using DAVID version 6.8 [30]. FDR <0.05 indicated significance.

### 2.5 Construction of a diagnostic model on basis of the fibroblast-related DEGs

On account of the expression levels of the overlapping DEGs, rms version 6.3-0 [31] in R 4.3.1 was used for univariate logistic regression analyses, as well as genes with *P* < 0.05 were retained. These selected genes were evaluated with the LASSO algorithm using the lars package version 1.2 in R 4.3.1 [32].

The optimized fibroblast-related DEGs were extracted from the combined expression profile dataset (training dataset), and their expression levels were displayed. The support vector machine (SVM) method [33] was applied to construct a classifier for disease diagnosis according to these optimized DEGs (core: Sigmoid Kernel; cross: 10-fold cross validation), and the efficacy of the diagnostic model was evaluated using the ROC curve method in pROC package version 1.12.1 of R 3.6.1 [27]. In addition, the keloid-related expression profile (GSE185309) was downloaded as the validation dataset, and the expression levels of the corresponding fibrosis-related genes were extracted. Finally, the ROC curve method was also employed to verify model efficacy using the validation dataset (GSE185309).

### 2.6 Correlations with immune cell types

GSVA version 1.36.3 in R 4.3.1 was employed to evaluate the proportions of immune cell types in the combined expression profile dataset [26]. Differences in the proportion distribution of each immune cell type between the keloid as well as control groups were assessed by Kruskal–Wallis test implemented in R 4.3.1. Finally, the correlations between the differentially abundant immune cells, as well as the fibroblast-related DEGs used for model construction were evaluated based on Pearson correlation coefficients using the cor function in R 4.3.1.

### 2.7 Real-time quantitative PCR (RT-qPCR)

The expression of the optimized fibroblast-related DEGs (*CCNB1*, *EGFR*, *E2F8*, *BTG1*, *TP63*, and *IGF1*) was further validated in human tissues using RT-qPCR. Samples from six patients with keloids were collected from the Plastic Surgery Hospital Chinese Academy of Medical Sciences (Beijing, China); as well as the keloid tissues and adjacent normal tissues were harvested. The study protocol was approved by the Ethics Committee of Plastic Surgery Hospital Chinese Academy of Medical Sciences, and informed consent was obtained from all participants.

Total RNA was isolated from the tissues using TRIzol reagent; as well as reverse-transcribed into cDNA using the PrimeScript 1st Strand cDNA Synthesis Kit (Takara, Kusatsu, Japan). The sequences of all primers were shown in Table 1, as well as *GAPDH* served as an internal control. The relative mRNA expression levels of the relevant genes were analyzed by the 2^−ΔΔCT^ method. The data are reported as the mean ± standard deviation; as well as the Student's *t*-test in GraphPad Prism 5 was utilized for comparisons between two groups. *P* < 0.05 was considered significant.

**Table 1:** Primer sequences for RT-qPCR assay.

Primer	Sequences (5′-3′)
GAPDH-hF	TGACAACTTTGGTATCGTGGAAGG
GAPDH-hR	AGGCAGGGATGATGTTCTGGAGAG
EGFR-hF	AGGCACGAGTAACAAGCTCAC
EGFR-hR	ATGAGGACATAACCAGCCACC
E2F8-hF	CCTGAGATCCGCAACAGAGAT
E2F8-hR	AGATGTCATTATTCACAGCAGGG
BTG1-hF	CCACCATGATAGGCGAGATCG
BTG1-hR	GGTTGATGCGAATACAACGGTA
TP63-hF	GGACCAGCAGATTCAGAACGG
TP63-hR	AGGACACGTCGAAACTGTGC
IGF1-hF	GCTCTTCAGTTCGTGTGTGGA
IGF1-hR	GCCTCCTTAGATCACAGCTCC
CCNB1-hF	AATAAGGCGAAGATCAACATGGC
CCNB1-hR	TTTGTTACCAATGTCCCCAAGAG

## 3 Results

### 3.1 Screen of fibroblast-related genes, and assessment of sample fibroblast scores

**Fig. 1:**
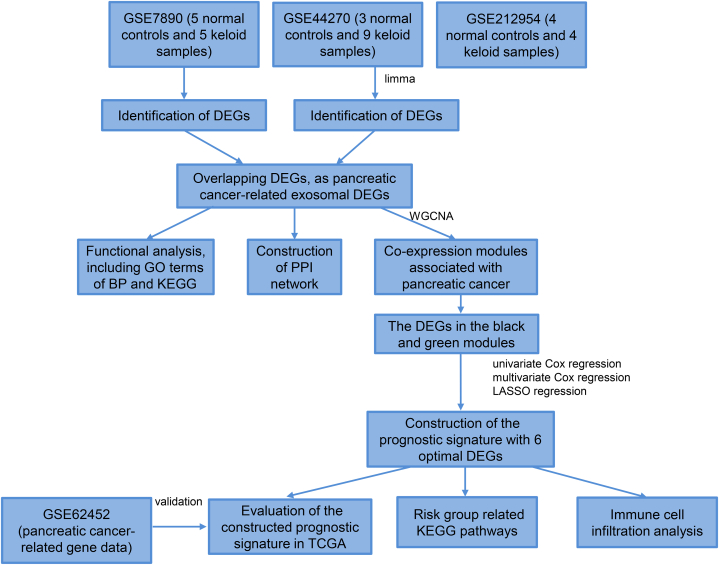
Workflow of the bioinformatics analysis.

**Fig. 2:**
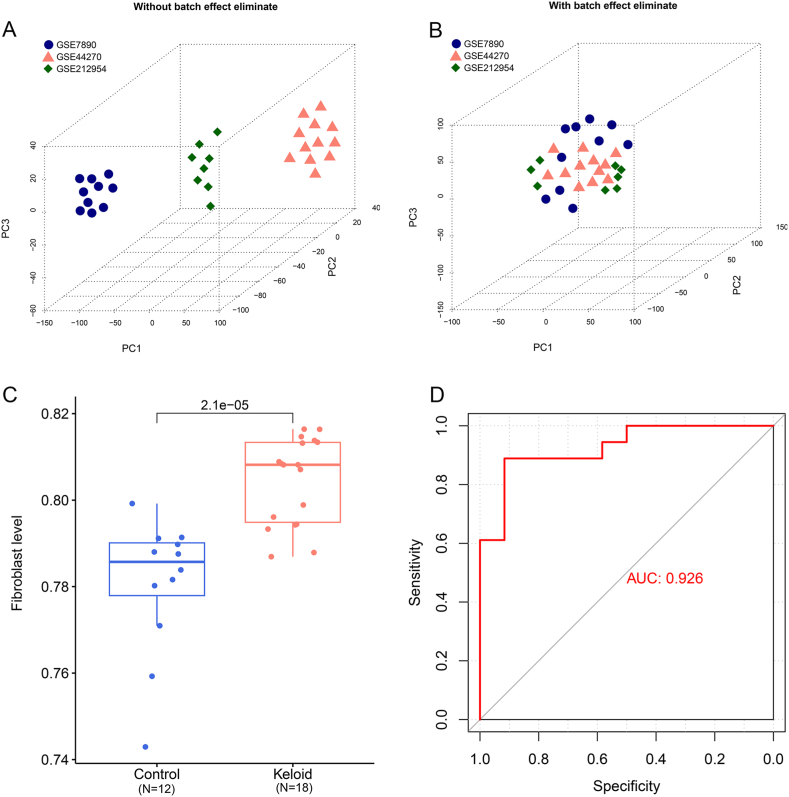
**Screening of fibroblast-related genes and assessment of sample fibroblast scores.** The distribution of all samples in the GSE7890, GSE44270, and GSE212954 datasets without (A) and with (B) batch effect elimination. Blue circles represent samples in the GSE7890, pink triangles represent the samples in GSE44270, and green rhombuses represent the samples in GSE2122954. (C) The fibroblast scores in the keloid groups were significantly higher than that in the control group. *P* = 2.1e-05. (D) An ROC curve analysis showed that the fibroblast score had a good ability to identify the sample type.

A workflow of the bioinformatics analyses used in this study is shown in Fig. 1. The samples in GSE7890, GSE44270, and GSE212954 before and after removing batch effects are shown in Fig. 2A and B. Based on the GSEA database, seven biological processes in GO analyses related to fibroblast apoptosis and proliferation were involved, and 124 fibroblast-related genes were obtained (after removing duplicates). Then, the fibroblast score for each sample was evaluated using GSVA, revealing that the fibroblast scores were significantly higher in the keloid group (*P* < 0.05) than those in the control group (Fig. 2C). Furthermore, the area under the curve (AUC) value in the ROC analysis was 0.926, suggesting the fibroblast score was a good way to identify the sample type (Fig. 2D).

### 3.2 Selection of genes related to keloid and fibroblast scores by a WGCNA

**Fig. 3:**
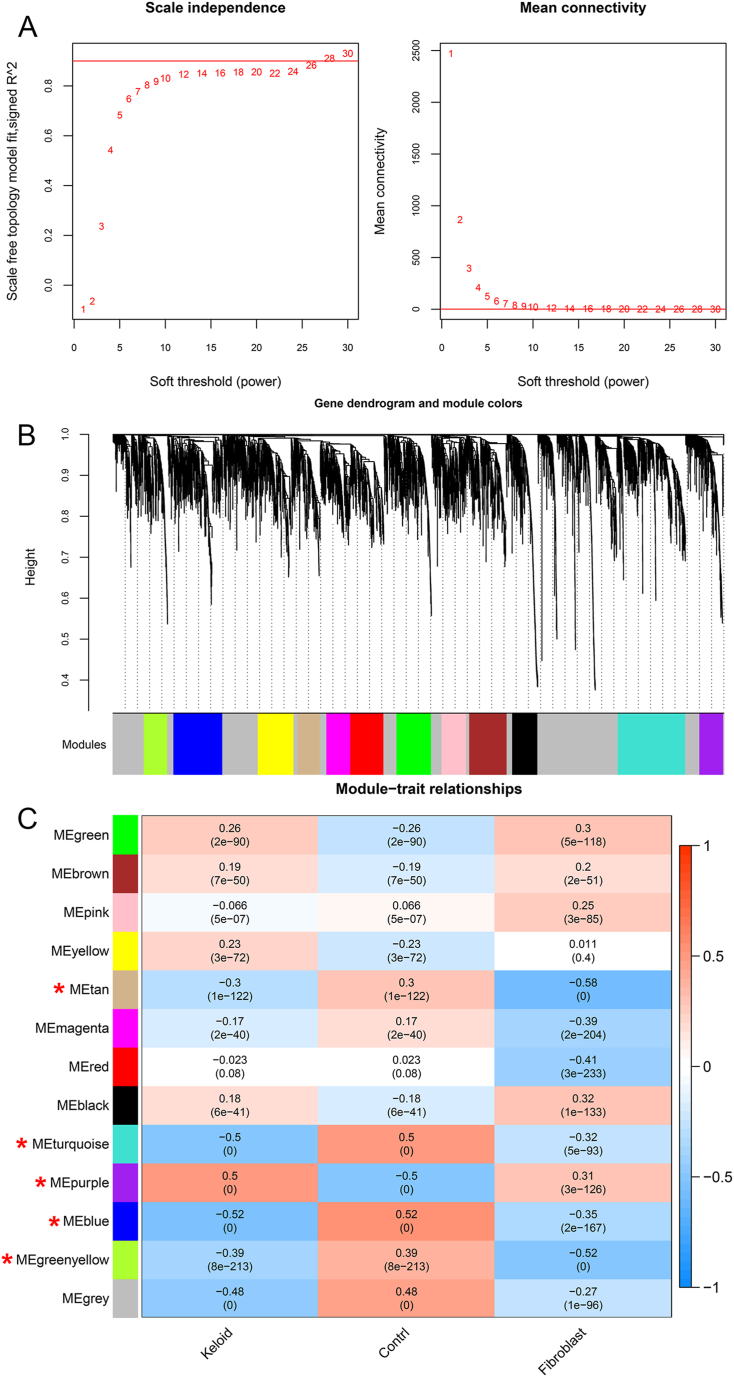
**Selection of genes related to keloids and fibroblast scores by WGCNA.** (A) Parameters for co-expression network construction. Left: the power selection diagram for the adjacency matrix weight parameter. The horizontal axis represents the weight parameter power, and the vertical axis represents the squared correlation coefficient between log(k) and log(p(k)) in the corresponding network. The red line indicates the standard line where the squared value of the correlation coefficient reaches 0.9. Right: Schematic diagram of the average connectivity degree of genes under different power parameters. The red line represents the value of the average connectivity degree of network nodes under the power parameter of the adjacency matrix weight. (B) Module partition tree diagram; 13 modules were obtained. Each color represents a different module. (C) Correlation heatmap between the 13 modules and the keloid or control groups or fibroblast scores.

The expression of all genes in the combined expression profile dataset were analyzed. In order to satisfy the premise of the scale-free network distribution as far as possible, the weight parameter power of the adjacency matrix was explored; network construction parameters were set and the scale-free distribution topology matrix was obtained. In Fig. 3A, the value of power when the square value of correlation coefficient reached 0.9 for the first time was selected (i.e., power = 28); for this power value, the average node connectivity of the constructed co-expression network was 1, which fully conformed to the properties of the small-world network. Then, the dissimilarity coefficient between nodes was calculated, as well as the system clustering tree was obtained with the minimum number of genes in each module ≥200 as well as the pruning height set to cutHeight = 0.995. In total, 13 modules were obtained (Fig. 3B). After that, the correlations among modules, fibroblast scores, as well as disease status were calculated (Fig. 3C). Among the 13 modules, five modules (blue, greenyellow, purple, tan, and turquoise) had significant correlations with both the disease state and fibroblast scores with absolute values of correlation coefficients ≥0.3; these modules contained 463, 222, 225, 213, and 637 genes, respectively. The genes related to the disease state and fibroblast score in these five modules were retained, including 1760 genes.

### 3.3 Identification of DEGs and functional analysis

**Fig. 4:**
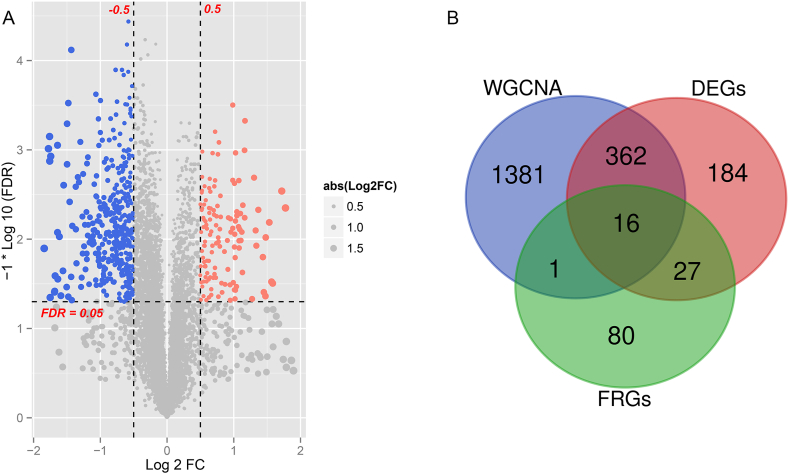
**Identification of differentially expressed genes (DEGs).** (A) Volcano plot of the identified DEGs. Blue and red dots indicate significantly down-regulated and up-regulated genes, respectively, and the black horizontal line indicates FDR <0.05. (B) Venn diagram of the DEGs, fibroblast-related genes, and genes obtained by WGCNA.

Based on FDR <0.05 as well as |log_2_ FC| > 0.5, 589 DEGs, including 440 down-regulated and 149 up-regulated genes, were identified in the keloid samples in comparison to the control samples (Fig. 4A). In a comparison with genes obtained by the WGCNA (1760), and 124 fibroblast-related genes, 16 overlapping genes were found, including tumor protein p53-inducible nuclear protein 1 (*TP53INP1*), E2F transcription factor 8 (*E2F8*), cyclin A2 (*CCNA2*), Bcl2-associated X protein (*BAX*), cyclin B1 (*CCNB1*), B-cell translocation gene 1 (*BTG1*), *CKS2*, fibroblast growth factor 10 (*FGF10*), collagen type III alpha 1 (*COL3A1*), cyclin-dependent kinase regulatory subunit 1B (*CKS1B)*, insulin-like growth factor 1 (*IGF1*), superoxide dismutase 2 (*SOD2*), platelet-derived growth factor subunit B (*PDGFB*), secreted frizzled-related protein 1 (*SFRP1*), epidermal growth factor receptor (*EGFR*), and *TP63* (Fig. 4B).

**Fig. 5:**
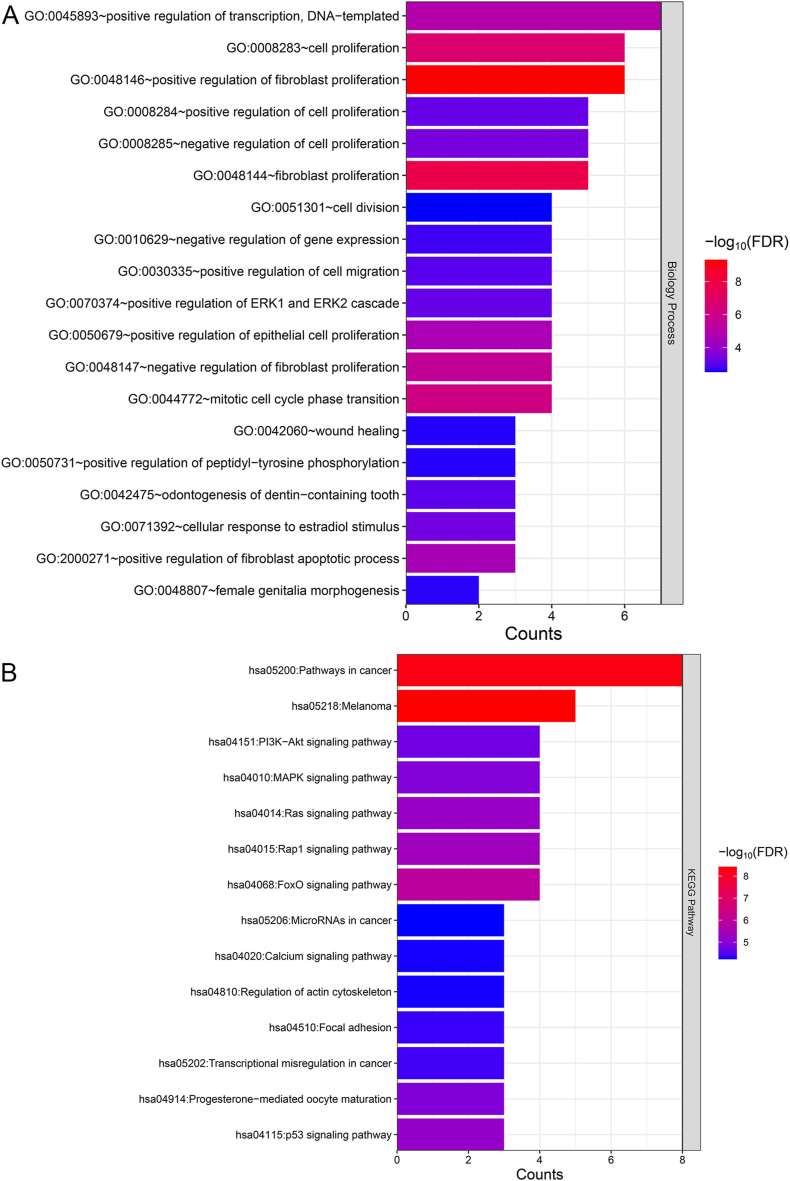
**Functional analysis of the overlapping DEGs.** (A) GO enrichment analysis of the overlapping DEGs. (B) KEGG enrichment analysis of the overlapping DEGs. The horizontal axis indicates the number of genes, the vertical axis indicates the item name, and the color indicates significance.

The 16 overlapping DEGs were further evaluated using GO and KEGG pathway analyses, revealing 19 significant GO terms in the biological process category and 14 KEGG pathways. According to the results of Fig. 5A, these genes were involved in the GO terms “positive regulation of ERK1 and ERK2 cascade,” “fibroblast proliferation,” “cell division,” “mitotic cell cycle phase transition,” and “wound healing.” Additionally, the overlapping DEGs were enriched in the following pathways: “Rap1 signaling pathway,” “FoxO signaling pathway,” “p53 signaling pathway,” “Ras signaling pathway,” “pathways in cancer,” “MAPK signaling pathway,” as well as “PI3K-Akt signaling pathway” (Fig. 5B).

### 3.4 Construction and evaluation of a diagnostic model

**Fig. 6:**
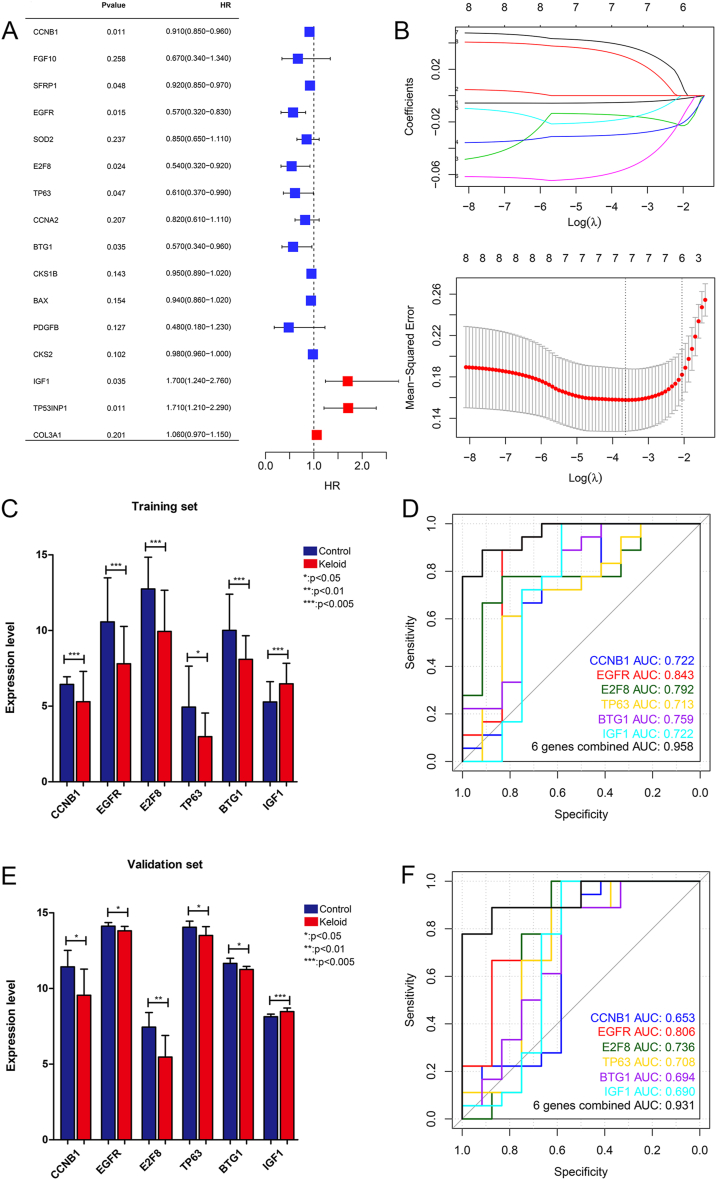
**Construction and evaluation of a diagnostic model.** (A) Eight genes with *P* < 0.05 identified by univariate logistic regression analyses based on the expression levels of 16 overlapping DEGs. (B) LASSO analysis identified six optimized DEGs. (C) Expression levels of the six optimized DEGs in the control and keloid samples in the combined training dataset. (D) ROC curves for each gene and the constructed diagnostic model using the combined training dataset. (E) Expression levels of the six optimized DEGs in the control and keloid samples in the validation dataset. (F) ROC curves for each gene and the constructed diagnostic model using the validation dataset. *P < 0.05, **P < 0.01, ***P < 0.005.

On basis of the expression of the aforementioned 16 overlapping fibroblast-related DEGs, univariate logistic regression analyses were performed. Eight genes with *P* < 0.05 were obtained, including *CCNB1*, *SERP1*, *EGFR*, *E2F8*, *TP63*, *BTG1*, *IGF1*, and *TP53INP1* (Fig. 6A). Following a LASSO regression analysis, six optimized DEGs were identified, i.e., *CCNB1*, *EGFR*, *E2F8*, *BTG1*, *TP63*, and *IGF1* (Fig. 6B).

The expression levels and efficiency of the identified six DEGs were analyzed in the combined training dataset and validation dataset. In the training dataset, the expression levels of *CCNB1*, *EGFR*, *E2F8*, *BTG1*, as well as *TP63* were remarkedly lower in the keloid samples than in the control samples (*P* < 0.05); however, the expression of *IGF1* was evidently enhanced in the keloid samples elative to the control samples (*P* < 0.05, Fig. 6C). Thereafter, a gene-based diagnostic model was constructed, and the efficacy of the model was evaluated. Using the combined training dataset, the AUC values for the six genes were all above 0.7, whereas the combined AUC for the six genes was 0.958 (Fig. 6D), indicating that the newly constructed diagnostic model based on the combination of the six genes had better diagnostic ability for keloids that those of each gene marker alone. Furthermore, the expression trends of the six genes in the different groups in the validation dataset were in keeping with those in the combined training dataset (Fig. 6E). The AUC values for the six genes were all above 0.65, whereas the combined AUC for the six genes was 0.931 in the validation dataset (Fig. 6F). The consistency in the results obtained using the combined training and validation datasets supported the reliability of the proposed diagnostic model.

### 3.5 Relationship between the optimized DEGs and immune cell types

**Fig. 7:**
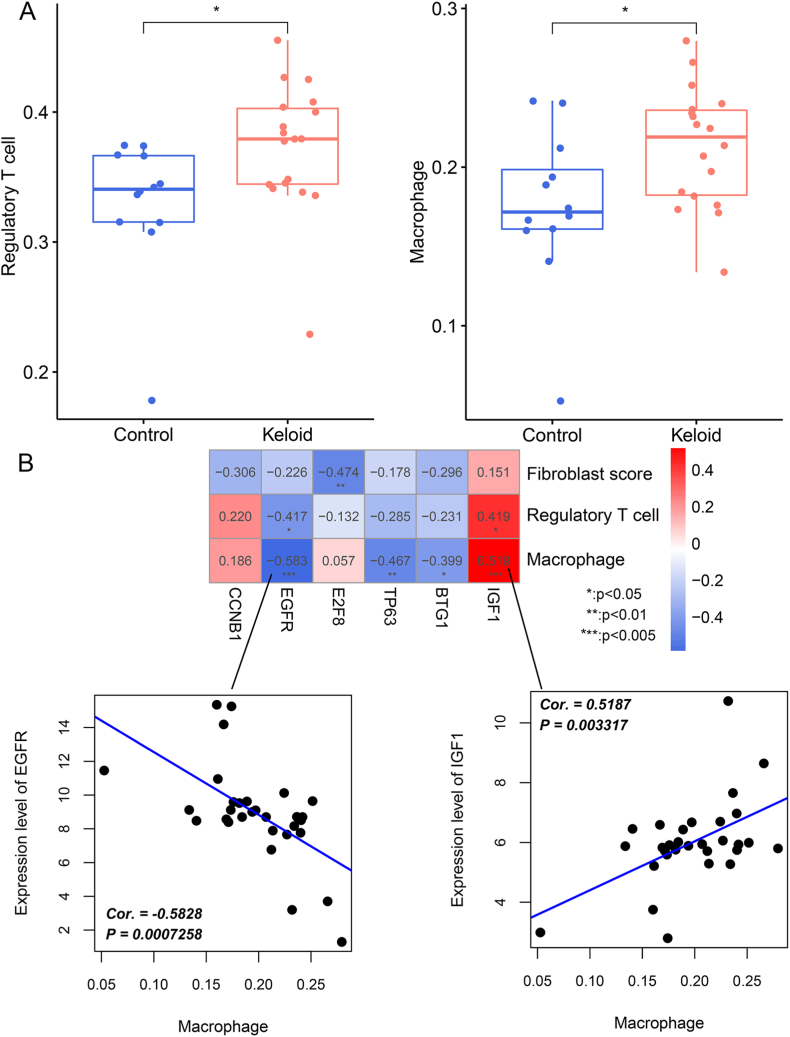
**Relationship between the optimized DEGs and immune cells.** (A) Two immune cell types (regulatory T cells and macrophages) with significant differences in relative frequences between control and keloid samples. (B) Correlations between the optimized DEGs and the two differential immune cell types or fibroblast scores. *P < 0.05, **P < 0.01, ***P < 0.005.

After comparing the differences in the proportion distribution of immune cells between the keloid as well as control groups, two immune cell types were screened out, regulatory T cells and macrophages. Their proportions in the keloid groups were evidently risen (*P* < 0.05) in comparison with the control group (Fig. 7A). In a correlation analysis of the optimized DEGs, fibroblast scores, and the two immune cells, *E2F8* was markedly negatively linked with the fibroblast score (cor. −0.474, *P* < 0.01); regulatory T cells had a significant negative correlation with *EGFR* (cor. −0.417, *P* < 0.05) as well as a significant positive correlation with *IGF1* (cor. 0.419, *P* < 0.05). In addition, macrophages had significantly positive correlation with *IGF1* (cor. 0.519, *P* < 0.005) as well as negative relationship with *EGFR* (cor. −0.583, *P* < 0.005), *TP63* (cor. −0.467, *P* < 0.01), as well as *BTG1* (cor. −0.399, *P* < 0.05) (Fig. 7B).

### 3.6 Verification of the expression of the optimized fibroblast-related DEGs by RT-qPCR

**Fig. 8:**
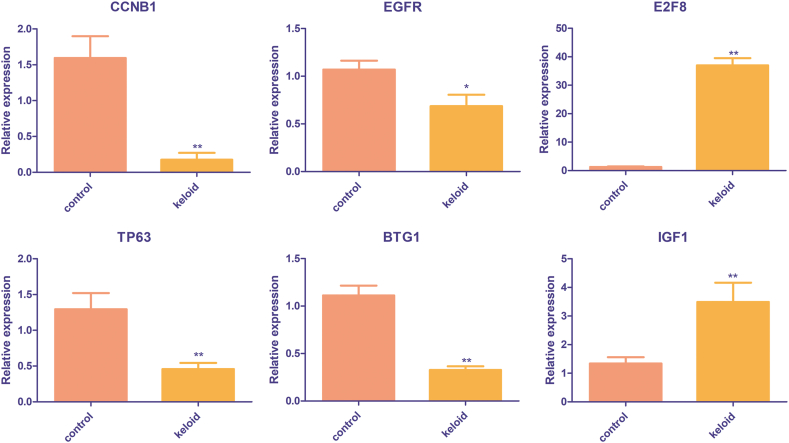
**Expression levels of the optimized fibroblast-related DEGs (*CCNB1*, *EGFR*, *E2F8*, *BTG1*, *TP63*, and *IGF1*) in human tissues.** *P < 0.05, **P < 0.01, compared with the control.

The expression levels of *CCNB1*, *EGFR*, *E2F8*, *BTG1*, *TP63*, and *IGF1* were further validated in human tissues. In comparison to levels in control tissues, the expression levels of *CCNB1*, *EGFR*, *BTG1*, as well as *TP63* were evidently lower in the keloid tissues (*P* < 0.05), while the expression levels of *E2F8* as well as *IGF1* were evidently higher in the keloid tissues (*P* < 0.05, Fig. 8). The outcomes exhibited the expression trends of *CCNB1*, *EGFR*, *BTG1*, *TP63*, and *IGF1* measured by RT-qPCR were in line with the results of the bioinformatics analysis (Fig. 6C–E). The proportion of consistent results between RT-qPCR as well as bioinformatics analysis was 83.33 %, indicating a relatively high reliability of our bioinformatics analysis.

## 4 Discussion

A keloids is a kind of benign fibrous tumor of the dermis, which poses a heavy psychological burden to patients [34]. Fibroblasts are key effector cells in the occurrence of keloids [13], as well as thus keloid fibroblast-related genes are potential therapeutic targets. In this study, 16 overlapping DEGs were identified; these genes were involved in various signaling pathways, containing the pathways of p53, MAPK, and PI3K-Akt; as well as pathways in cancer. Finally, fibroblast-related gene signature including genes (*CCNB1*, *EGFR*, *E2F8*, *BTG1*, *TP63*, and *IGF1*) was used for diagnostic model construction. An ROC curve revealed that the model showed high sensitivity and specificity (based on AUC values). Additionally, regulatory T cells and macrophages showed higher relative frequencies in keloid tissues than in controls.

A recent study has shown that the growth characteristics of keloids may be attributed to an imbalance between rates of proliferation and apoptosis [35]. The MAPK signaling pathway is the classic pathway related to cell proliferation [36]. The p53 tumor suppressor has a central effect on cell apoptosis [37]. Similarly, PI3K-Akt signaling pathway is also related with the mediation of cell proliferation, differentiation, as well as apoptosis [38]. Of note, an imbalance between cell proliferation and apoptosis is also a feature of carcinogenesis. In this study, a pathway enrichment analysis revealed cancer-related pathways and microRNAs. In fact, keloids can exhibit various cancer-like features, for example, extremely high recurrence rates as well as uncontrolled progressive growth [39]. There is increasing evidence for interactions between pro-tumor factors and suppressors, which may explain its aggressive clinical behaviors. Moreover, the most striking similarities between keloids as well as cancer are their shared epithelial-mesenchymal transition, cellular bioenergetics, as well as epigenetic methylation signatures [40]. We speculated that these proliferation-, apoptosis-, and cancer-related pathways contribute to the progression of keloids. However, the specific mechanism needs to be further explored.

Based on the six fibroblast-related signature genes (*CCNB1*, *EGFR*, *E2F8*, *BTG1*, *TP63*, and *IGF1*), a diagnostic model was developed in this study. *CCNB1* was enriched in cell cycle-related functions and the p53 signaling pathway. Keloids are featured by dermal germination beyond the original margin of the wound, depending on the ectopic cell cycle [34]. For p53, compaed to the normal scar tissues, it expression was often higher [41]. Importantly, Wang et al. [42] have also reported that changes in *CCNB1* may be involved in keloid formation by regulating cell cycle and p53 signaling pathway. Huang et al. [43] demonstrated *CCNB1* expression was decreased in keloid fibroblasts treated by 5-fluorouracil, suggesting that CCNB1 may serve as a treatment target for keloids. In addition to *CCNB1*, *IGF1* is involved in the p53 signaling pathway, and may play functions in the pathogenesis of keloids. EGFR can activate the tyrosine protein kinase-binding domain of the receptor when bound to its corresponding ligand EGF, which further activates downstream signaling pathways, thereby causing cell division and proliferation [44]. It has been reported that EGFR is down-regulated in keloid tissues [45], consistent with our results. EGF-induced fibroblasts in keloid tissues show lower mobility than those of fibroblasts in normal tissues and undergo reduced mitosis [46]. Therefore, the down-regulation of EGFR may promote the development of keloids. Both *E2F8* and *BTG1* were associated with cell proliferation. *TP63* and *IGF1* were also involved in some cancer-related pathways. To our best knowledge, the effects of these four genes on keloids have not been investigated. These fibroblast-related genes may contribute to the cancer-like features of keloids. ROC curves revealed that the AUC values of the six genes were all above 0.7, whereas the AUC value for the combination of the six genes was 0.958, supporting the high predictive value of the model [47,48]. Taken together, we can infer that these six genes are candidate genes in keloids, and the model based on these six genes can be used for diagnosis. However, the exact roles of these genes need to be further studied.

In addition, infiltration of immune cells is a hallmark of keloid tissues; as well as preferential collection of immune cells regulates skin repair process through interactions with keloid fibroblasts [49]. There is increasing recognition of the abnormal immune cell composition as well as activity in nonlesional skin of patients with keloids [50,51]. Our research observed regulatory T cells and macrophages have higher relative frequencies in keloid tissues than in controls. Studies have illustrated the number of regulatory T cells is increased in the lesions of keloid skin [52,53]. These cells promote the preferential accumulation of type III collagen in the presence of anti-CD3/CD28 [52]. Importantly, the accumulation of regulatory T cells at sites of injury can regulate the polarization of M1 to M2 macrophages [54]. The feature of the common wound healing process is an orderly interim from a M1 macrophages-dominated inflammatory phase at early stage to a M2 macrophages-dominated recovery phase [55]. Disruption of this progression can contribute to the prolonged inflammation, delayed wound healing or increased scarring. The proportion of M2 macrophages is abnormally elevated in keloid lesions [56,57], consistent with our findings. Specially, the two kinds of immune cells were significantly negatively linked to *EGFR* and *TP63*; as well as positively associated with *IGF1*. Nevertheless, detailed relationships between different types of immune cells as well as fibroblast-related genes are still necessary to beinvestiagted.

This study had some limitations. For example, the samples size was small, and further experiments using a larger sample size are needed. Additionally, our study was based on bioinformatics analyses; in vitro and in vivo experiments are warranted to further explore specific roles and mechanisms of actions of the six optimized DEGs and two immune cell types in keloid development.

## 5 Conclusion

A fibroblast-related diagnostic model for keloid based on a six-gene signature (*CCNB1*, *EGFR*, *E2F8*, *BTG1*, *TP63*, and *IGF1*) was proposed, showing high predictive accuracy in keloid diagnosis. Furthermore, our results supported the critical roles of regulatory T cells and macrophages in keloid formation. All the findings provide a basis for the early diagnosis as well as control of keloids, thereby improving clinical outcomes.

## Ethical statement

The research protocol was approved by the Ethics Committee of Plastic Surgery Hospital Chinese Academy of Medical Sciences, and informed consent was obtained from all participants.

## Data availability statement

The data analyzed in this study was available from the Gene Expression Omnibus (GEO) database, including GSE7890, GSE44270, and GSE212954.

## Funding

None.

## CRediT authorship contribution statement

**Yue Qi:** Writing – original draft, Methodology, Data curation, Conceptualization. **GuiE. Ma:** Writing – review & editing, Resources, Project administration, Formal analysis.

## Declaration of competing interest

The authors declare that they have no known competing financial interests or personal relationships that could have appeared to influence the work reported in this paper.

## References

[1] Naik P.P. (2022). Novel targets and therapies for keloid. Clin. Exp. Dermatol..

[2] Ogawa R. (2017). Keloid and hypertrophic scars are the result of chronic inflammation in the reticular dermis. Int. J. Mol. Sci..

[3] Chao H., Zheng L., Hsu P. (2023). IL-13RA2 downregulation in fibroblasts promotes keloid fibrosis via JAK/STAT6 activation. JCI Insight.

[4] Lee J.Y., Yang C.C., Chao S.C. (2004). Histopathological differential diagnosis of keloid and hypertrophic scar. Am. J. Dermatopathol..

[5] van Leeuwen M.C., Stokmans S.C., Bulstra A.E. (2015). Surgical excision with adjuvant irradiation for treatment of keloid scars: a systematic review. Plast Reconstr Surg Glob Open.

[6] Monika P., Waiker P.V., Chandraprabha M.N. (2021).

[7] Henderson N.C., Rieder F., Wynn T.A. (2020).

[8] Liu X., Chen W., Zeng Q. (2022). Single-cell RNA-sequencing reveals lineage-specific regulatory changes of fibroblasts and vascular endothelial cells in keloids. J. Invest. Dermatol..

[9] Rodrigues M., Kosaric N., Bonham C.A. (2019). Wound healing: a cellular perspective. Physiol. Rev..

[10] Shaw T.J., Martin P. (2016). Wound repair: a showcase for cell plasticity and migration. Curr. Opin. Cell Biol..

[11] Hinz B., Lagares D. (2020).

[12] Feng Y., Wu J.J., Sun Z.L. (2020). Targeted apoptosis of myofibroblasts by elesclomol inhibits hypertrophic scar formation. EBioMedicine.

[13] Zhou P., Shi L., Li Q. (2015). Overexpression of RACK1 inhibits collagen synthesis in keloid fibroblasts via inhibition of transforming growth factor-β1/Smad signaling pathway. Int. J. Clin. Exp. Med..

[14] Macarak E.J., Wermuth P.J., Rosenbloom J. (2021). Keloid disorder: fibroblast differentiation and gene expression profile in fibrotic skin diseases. Exp. Dermatol..

[15] He S., Liu X., Yang Y. (2010). Mechanisms of transforming growth factor beta(1)/Smad signalling mediated by mitogen-activated protein kinase pathways in keloid fibroblasts. Br. J. Dermatol..

[16] Brown J.J., Ollier W., Arscott G. (2008). Genetic susceptibility to keloid scarring: SMAD gene SNP frequencies in Afro-Caribbeans. Exp. Dermatol..

[17] Lee W.J., Choi I.K., Lee J.H. (2013). A novel three-dimensional model system for keloid study: organotypic multicellular scar model. Wound Repair Regen..

[18] Marneros A.G. (2019). A role for the E3 ubiquitin ligase NEDD4 in keloid pathogenesis. J. Invest. Dermatol..

[19] Fujita M., Yamamoto Y., Jiang J.J. (2019). NEDD4 is involved in inflammation development during keloid formation. J. Invest. Dermatol..

[20] Nangole F.W., Agak G.W. (2019). Keloid pathophysiology: fibroblast or inflammatory disorders?. JPRAS Open.

[21] Shan M., Wang Y. (2022). Viewing keloids within the immune microenvironment. Am J Transl Res.

[22] Smith J.C., Boone B.E., Opalenik S.R. (2008). Gene profiling of keloid fibroblasts shows altered expression in multiple fibrosis-associated pathways. J. Invest. Dermatol..

[23] Hahn J.M., Glaser K., McFarland K.L. (2013). Keloid-derived keratinocytes exhibit an abnormal gene expression profile consistent with a distinct causal role in keloid pathology. Wound Repair Regen..

[24] Leek J.T., Johnson W.E., Parker H.S. (2012). The sva package for removing batch effects and other unwanted variation in high-throughput experiments. Bioinformatics.

[25] Ye L., Zhang T., Kang Z. (2019). Tumor-infiltrating immune cells act as a marker for prognosis in colorectal cancer. Front. Immunol..

[26] Na K., Lkhagva-Yondon E., Kim M. (2020). Oral treatment with Aloe polysaccharide ameliorates ovalbumin-induced atopic dermatitis by restoring tight junctions in skin. Scand. J. Immunol..

[27] Robin X., Turck N., Hainard A. (2011). pROC: an open-source package for R and S+ to analyze and compare ROC curves. BMC Bioinf..

[28] Langfelder P., Horvath S. (2008). WGCNA: an R package for weighted correlation network analysis. BMC Bioinf..

[29] Ritchie M.E., Phipson B., Wu D. (2015). Limma powers differential expression analyses for RNA-sequencing and microarray studies. Nucleic Acids Res..

[30] Huang da W., Sherman B.T., Lempicki R.A. (2009). Systematic and integrative analysis of large gene lists using DAVID bioinformatics resources. Nat. Protoc..

[31] Yang H., Zhou J., Pan H. (2021). Mesenchymal stem cells derived-exosomes as a new therapeutic strategy for acute soft tissue injury. Cell Biochem. Funct..

[32] Goeman J.J. (2010). L1 penalized estimation in the Cox proportional hazards model. Biom. J..

[33] Ju R., Wu W., Tang Q. (2015). Association analysis between the polymorphisms of HSD11B1 and H6PD and risk of polycystic ovary syndrome in Chinese population. PLoS One.

[34] Fong C.Y., Biswas A., Subramanian A. (2014). Human keloid cell characterization and inhibition of growth with human Wharton's jelly stem cell extracts. J. Cell. Biochem..

[35] Zhang M.Z., Dong X.H., Zhang W.C. (2023). A comparison of proliferation levels in normal skin, physiological scar and keloid tissue. J Plast Surg Hand Surg..

[36] Meng X., Shi Y., Xiang X. (2020). Influence of miR-101 on proliferation of liver cancer cells through the MAPK/ERK signaling pathway. Oncol. Lett..

[37] Ingaramo M.C., Sánchez J.A., Dekanty A. (2018). Regulation and function of p53: a perspective from Drosophila studies. Mech. Dev..

[38] Li G., Li Y.Y., Sun J.E. (2016). ILK-PI3K/AKT pathway participates in cutaneous wound contraction by regulating fibroblast migration and differentiation to myofibroblast. Lab. Invest..

[39] Kimura K., Inadomi T., Yamauchi W. (2014). Dermatofibrosarcoma protuberans on the chest with a variety of clinical features masquerading as a keloid: is the disease really protuberant?. Ann. Dermatol..

[40] Tan S., Khumalo N., Bayat A. (2019). Understanding keloid pathobiology from a quasi-neoplastic perspective: less of a scar and more of a chronic inflammatory disease with cancer-like tendencies. Front. Immunol..

[41] Tanaka A., Hatoko M., Tada H. (2004). Expression of p53 family in scars. J. Dermatol. Sci..

[42] Wang H., Quan L., Liang J. (2017). Gene expression profiling analysis of keloids with and without hydrocortisone treatment. Exp. Ther. Med..

[43] Huang L., Wong Y.P., Cai Y.J. (2010). Low-dose 5-fluorouracil induces cell cycle G2 arrest and apoptosis in keloid fibroblasts. Br. J. Dermatol..

[44] Marmor M.D., Skaria K.B., Yarden Y. (2004). Signal transduction and oncogenesis by ErbB/HER receptors. Int. J. Radiat. Oncol. Biol. Phys..

[45] Satish L., Babu M., Tran K.T. (2004). Keloid fibroblast responsiveness to epidermal growth factor and activation of downstream intracellular signaling pathways. Wound Repair Regen..

[46] Kang S.U., Kim Y.S., Kim Y.E. (2017). Opposite effects of non-thermal plasma on cell migration and collagen production in keloid and normal fibroblasts. PLoS One.

[47] Jiang S., Zhang W., Lu Y. (2022). Development and validation of novel inflammatory response-related gene signature for sepsis prognosis. J. Zhejiang Univ. - Sci. B.

[48] Zhang H., Xia P., Liu J. (2021). ATIC inhibits autophagy in hepatocellular cancer through the AKT/FOXO3 pathway and serves as a prognostic signature for modeling patient survival. Int. J. Biol. Sci..

[49] Larouche J., Sheoran S., Maruyama K. (2018). Immune regulation of skin wound healing: mechanisms and novel therapeutic targets. Adv. Wound Care.

[50] Diaz A., Tan K., He H. (2020 Apr). Keloid lesions show increased IL-4/IL-13 signaling and respond to Th2-targeting dupilumab therapy. J. Eur. Acad. Dermatol. Venereol..

[51] Wu J., Del Duca E., Espino M. (2020). RNA sequencing keloid transcriptome associates keloids with Th2, Th1, Th17/Th22, and JAK3-skewing. Front. Immunol..

[52] Chen Y., Jin Q., Fu X. (2019). Connection between T regulatory cell enrichment and collagen deposition in keloid. Exp. Cell Res..

[53] Jin Q., Gui L., Niu F. (2018). Macrophages in keloid are potent at promoting the differentiation and function of regulatory T cells. Exp. Cell Res..

[54] Villalta S.A., Rosenthal W., Martinez L. (2014). Regulatory T cells suppress muscle inflammation and injury in muscular dystrophy. Sci. Transl. Med..

[55] Hesketh M., Sahin K.B., West Z.E. (2017). Macrophage phenotypes regulate scar formation and chronic wound healing. Int. J. Mol. Sci..

[56] Feng C., Shan M., Xia Y. (2022). Single-cell RNA sequencing reveals distinct immunology profiles in human keloid. Front. Immunol..

[57] Xia Y., Wang Y., Xiao Y. (2022). Identification of a diagnostic signature and immune cell infiltration characteristics in keloids. Front. Mol. Biosci..

