# Supplementary material for: Comprehensive bioinformatic analysis reveals a fibroblast-related gene signature for the diagnosis of keloids
Source: Heliyon. 2024 Jul 22;10(15):e35011. doi: 10.1016/j.heliyon.2024.e35011 (PMC11327581; doi:10.1016/j.heliyon.2024.e35011)
Supplement: Multimedia component 1 [file mmc1.doc]

1. Merge of data by sva

library(sva)

cdata <- read.table("input.txt", header = T, sep = "\t")

cdata=as.matrix(cdata[,-1])

csif <- read.table("batch.txt", header = T, sep = "\t")

modcombat = model.matrix(~1, data = csif)

batch = csif$batch

combat_edata = ComBat(dat=cdata,batch=batch,mod=modcombat,par.prior=TRUE,prior.plots=TRUE)

write.table(combat_edata,file="combat_edata.txt",sep="\t")

2.Evaluation of ssGSEA

library(GSVA)

rm(list=ls())

library(tidyverse)

options(stringsAsFactors = F)

geneSet <- read.csv("d:/reference.txt",header = F,sep = "\t",)

class(geneSet)

geneSet <- geneSet %>%

column_to_rownames("V1")%>%t()

a <- geneSet

a <- a[1:nrow(a),]

set <- colnames(a)

l <- list()

#i <- "Activated CD8 T cell"

for (i in set) {

x <- as.character(a[,i])

x <- x[nchar(x)!=0]

x <- as.character(x)

l[[i]] <-x

}

save(l,file = "d:/gene_set.Rdata")

logTPM=read.table("d:/combat_edata.txt",header=T)

save(logTPM,file = "d:/logTPM.Rdata")

load(file = "d:/logTPM.Rdata")

load(file = "d:/gene_set.Rdata")

dat <- as.matrix(logTPM[,-1])

ssgsea<- gsva(dat,l,method='ssgsea',kcdf='Gaussian',abs.ranking=TRUE)

ssgsea.1 <- ssgsea

for (i in colnames(ssgsea)) {

#i <- colnames(ssgsea)[1]

ssgsea.1[,i] <- (ssgsea[,i] -min(ssgsea[,i]))/(max(ssgsea[,i] )-min(ssgsea[,i] ))

}

ROC curves

library(pROC)

A=read.table("d:/immune.txt",header=T,sep="\t")

x <- A[,m:n]

y <- A[,a]

model <- lm(x, y)

pred <- predict(model, x)

pred <- fitted(model)

model <- svm(x, y)

modelroc <- roc(y,pred)

plot(modelroc, print.auc=TRUE, auc.polygon=TRUE, grid=c(0.1, 0.2),grid.col=c("black", "black"), max.auc.polygon=FALSE,auc.polygon.col="white", print.thres=TRUE,lwd=2)

3.WGCNA

library(WGCNA)

library(flashClust)

datExprA1p<-read.table("combat_edata.txt",sep="\t",head=F)

rownames(datExprA1p)<-datExprA1p[,1]

datExprA1p<-datExprA1p[-1,-1]

datExprA1p<-as.matrix(datExprA1p)

mode(datExprA1p)<-"numeric"

powers = c(c(1:10), seq(from = 12, to=30, by=2))

sft = pickSoftThreshold(t(datExprA1p), powerVector = powers, verbose = 5)

sizeGrWindow(9, 5)

par(mfrow = c(1,2));

cex1 =0.7;

plot(sft$fitIndices[,1], -sign(sft$fitIndices[,3])*sft$fitIndices[,2],xlab="Soft threshold (power)",ylab="Scale free topology model fit,signed R^2",type="n",main = paste("Scale independence"),cex.axis=0.7,cex.lab=0.9,cex.main=1.0);

text(sft$fitIndices[,1], -sign(sft$fitIndices[,3])*sft$fitIndices[,2],labels=powers,cex=cex1,col="red");

abline(h=0.9,col="red")

plot(sft$fitIndices[,1], sft$fitIndices[,5],xlab="Soft threshold (power)",ylab="Mean connectivity", type="n",main = paste("Mean connectivity"),cex.axis=0.7,cex.lab=0.9,cex.main=1.0)

text(sft$fitIndices[,1], sft$fitIndices[,5], labels=powers, cex=cex1,col="red")

abline(h=1,col="red")

softPower=9

datExprA1g = datExprA1p;dim(datExprA1g)

adjacencyA1 = adjacency(t(datExprA1g),power=softPower,type="signed");

diag(adjacencyA1)=0

dissTOMA1 = 1-TOMsimilarity(adjacencyA1, TOMType="signed")

geneTreeA1 = flashClust(as.dist(dissTOMA1), method="average")

mColorh=NULL

for (ds in 0:3){

tree = cutreeHybrid(dendro = geneTreeA1, pamStage=FALSE,

minClusterSize =100, cutHeight = 0.995,

deepSplit = ds, distM = dissTOMA1)

mColorh=cbind(mColorh,labels2colors(tree$labels));

}

plotDendroAndColors(geneTreeA1, mColorh, paste("dpSplt =",0:3), main = "",dendroLabels=FALSE);

modulesA1 = mColorh[,4]

plotDendroAndColors(geneTreeA1, modulesA1, "Modules", dendroLabels=FALSE, hang=0.03, addGuide=TRUE,

guideHang=0.05, main="Gene dendrogram and module colors",cex.main=0.8,cex.axis=0.7,cex.lab=0.9)

PCs1A = moduleEigengenes(t(datExprA1g), colors=modulesA1)

ME_1A = PCs1A$eigengenes

distPC1A = 1-abs(cor(ME_1A,use="p"))

distPC1A = ifelse(is.na(distPC1A), 0, distPC1A)

pcTree1A = hclust(as.dist(distPC1A),method="a")

MDS_1A = cmdscale(as.dist(distPC1A),2)

colorsA1 = names(table(modulesA1))

geneModuleMembership1 = signedKME(t(datExprA1g), ME_1A)

colnames(geneModuleMembership1)=paste("PC",colorsA1,".cor",sep="");

MMPvalue1=corPvalueStudent(as.matrix(geneModuleMembership1),dim(datExprA1g)[[2]]);

colnames(MMPvalue1)=paste("PC",colorsA1,".pval",sep="");

Gene = rownames(datExprA1g)

kMEtable1 = cbind(Gene,Gene,modulesA1)

for (i in 1:length(colorsA1))

kMEtable1 = cbind(kMEtable1, geneModuleMembership1[,i], MMPvalue1[,i])

colnames(kMEtable1)=c("PSID","Gene","Module",sort(c(colnames(geneModuleMembership1),colnames(MMPvalue1))))

write.csv(kMEtable1,"kMEtable1.csv",row.names=FALSE)

datTraits=read.table("trait.txt",header=T,sep="\t")

nGenes = ncol(datExprA1g);

nSamples = nrow(datExprA1g);

PCs1A = moduleEigengenes(t(datExprA1g), colors=modulesA1)

ME_1A = PCs1A$eigengenes

MEs = orderMEs(ME_1A)

moduleTraitCor =cor(MEs, datTraits, use = "p");

moduleTraitPvalue = corPvalueStudent(moduleTraitCor, nSamples);

textMatrix = paste(signif(moduleTraitCor, 2), "\n(",signif(moduleTraitPvalue, 1), ")", sep = "");

par(mar = c(6, 8.5, 3, 3));

dim(textMatrix) = dim(moduleTraitCor)

labeledHeatmap(Matrix =moduleTraitCor,xLabels = names(datTraits),yLabels = names(MEs),ySymbols = names(MEs),colorLabels = FALSE,colors = blueWhiteRed(50),textMatrix = textMatrix,setStdMargins = FALSE,cex.text = 0.7,cex.lab.x=0.8,cex.lab.y=0.8,zlim = c(-1,1),main = paste("Module-trait relationships"),cex.main=1)

4. Identification of DEGs using limma

library(limma)

data<-read.table("combat_edata.txt",sep = "\t",header=T,row.names=1)

data<- as.matrix(data)

mode(data)

group<-c(rep(CTRL,12),rep(KD,18))

design <- model.matrix(~ -1+factor(group))

colnames(design)<-c("CTRL","KD")

design

contrast.matrix <- makeContrasts(KD-CTRL,levels=design)

fit <- lmFit(data, design)

fit1 <- contrasts.fit(fit, contrast.matrix)

fit2 <- eBayes(fit1)

dif <- topTable(fit2, coef = 1, n = nrow(fit2), lfc = 0)

write.table(dif, "test.dif.txt", row.names = TRUE, sep = "\t")

5.Construction of a diagnostic model

Univariate logistic regression

library(plyr)

library(rms)

library(epiDisplay)

A=read.table("input.txt",header=T,sep="\t")

glm1<- glm(A[,1]~A[,N],data=A,family = binomial)

glm2<- summary(glm1);glm2

OR<-round(exp(coef(glm1)),2)

SE<-glm2$coefficients[,2]

CI5<-round(exp(coef(glm1)-1.96*SE),2)

CI95<-round(exp(coef(glm1)+1.96*SE),2)

CI<-paste0(CI5,'-',CI95)

P<-round(glm2$coefficients[,4],3)

res1<-data.frame(OR,CI,P)[-1,];res1

summary(glm1)

LASSO

library(glmnet)

data=read.table("input.txt",header=T)

x <- as.matrix(data[,a:b])

y <- data[,1]

alpha1_fit <- glmnet(x,y,alpha=1,family="gaussian")

plot(alpha1_fit,xvar="lambda",label=TRUE)

alpha1.fit <- cv.glmnet(x,y,type.measure = "mse",alpha=1,family="gaussian")

plot(alpha1.fit)

6. Construction of a SVM model and ROC analysis

library(e1071)

library(pROC)

A=read.table("input.txt",header=T,sep="\t")

x <- A[,N]

y <- A[,1]

model <- svm(x, y)

pred <- predict(model, x)

pred <- fitted(model)

model <- svm(x, y)

modelroc <- roc(y,pred)

plot(modelroc, print.auc=TRUE, auc.polygon=TRUE, grid=c(0.1, 0.2),grid.col=c("grey", "grey"), max.auc.polygon=FALSE,auc.polygon.col="white", print.thres=TRUE,lwd=2)

7. Assessment of immune by ssGSEA

library(GSVA)

rm(list=ls())

library(tidyverse)

options(stringsAsFactors = F)

geneSet <- read.csv("immune-reference.txt",header = F,sep = "\t",)

class(geneSet)

geneSet <- geneSet %>%

column_to_rownames("V1")%>%t()

a <- geneSet

a <- a[1:nrow(a),]

set <- colnames(a)

l <- list()

#i <- "Activated CD8 T cell"

for (i in set) {

x <- as.character(a[,i])

x <- x[nchar(x)!=0]

x <- as.character(x)

l[[i]] <-x

}

save(l,file = "gene_set.Rdata")

logTPM=read.table("input.txt",header=T)

save(logTPM,file = "logTPM.Rdata")

load(file = "logTPM.Rdata")

load(file = "gene_set.Rdata")

dat <- as.matrix(logTPM[,-1])

ssgsea<- gsva(dat,l,method='ssgsea',kcdf='Gaussian',abs.ranking=TRUE)

ssgsea.1 <- ssgsea

for (i in colnames(ssgsea)) {

#i <- colnames(ssgsea)[1]

ssgsea.1[,i] <- (ssgsea[,i] -min(ssgsea[,i]))/(max(ssgsea[,i] )-min(ssgsea[,i] ))

}

8. Correlation analysis

A=read.table("input.txt",header=T,sep="\t")

res2=rcorr(as.matrix(A[,-1]))

write.table(res2$P,file="p.txt",sep="\t")

write.table(res2$r,file="r.txt",sep="\t")
