# Supplementary material for: Comprehensive bioinformatic analysis reveals a fibroblast-related gene signature for the diagnosis of keloids
Source: Heliyon. 2024 Jul 22;10(15):e35011. doi: 10.1016/j.heliyon.2024.e35011 (PMC11327581; doi:10.1016/j.heliyon.2024.e35011)
Supplement: Multimedia component 2 [file mmc2.pdf]

797601503589171200_MaGuiE_Comprehensive bioinformatic analysis reveals a fibroblast-related gene signature for the diagnosis of keloids.docx

by a15a15

Submission date: 12-Jul-2024 03:54AM (UTC-0400)

Submission ID: 2415617828

File name: 797601503589171200_MaGuiE_Comprehensive bioinformatic analysis reveals a fibroblast-related gene signature for the diagnosis of keloids.docx (109.21K)

Word count: 4045

Character count: 22741

Comprehensive bioinformatic analysis reveals a fibroblast-related gene signature for the diagnosis of keloids

Running title: Diagnostic model in keloids

Highlights:

1. *CCNB1*, *EGFR*, *E2F8*, *BTG1*, *TP63*, and *IGF1* were associated with keloids.
2. A diagnostic model based on the signature genes showed good performance.
3. Proportions of regulatory T cells and macrophages were high in keloid tissues.

Abstract

Aim: A keloid is a fibroproliferative cutaneous disorder secondary to skin injury, caused by an imbalance in fibroblast proliferation and apoptosis. However, the pathogenesis is not fully understood. In this study, candidate genes for keloid were identified and used to construct a diagnostic model.

Methods: Three datasets related to keloids were downloaded from NCBI Gene Expression Omnibus. Fibroblast-related genes were screened, and fibroblast scores for the samples were determined. Then, a weighted gene co-expression network analysis (WGCNA) was used to identify modules and genes associated with keloids and the fibroblast score. Differentially expressed genes (DEGs) between keloid and control samples were identified and compared with fibroblast-related genes and genes in the modules. Overlapping genes were evaluated using functional enrichment analyses. Signature genes were further screened, and a diagnostic model was constructed. Finally, correlations between immune cell frequences and signature genes were analyzed.

Results: In total, 124 fibroblast-related genes were obtained, and the fibroblast score was an effective indicator of the sample type. WGCNA revealed five modules that were significantly correlated with both the disease state and fibroblast scores, including 1760 genes. Additionally, 589 DEGs were identified, including 16 that overlapped with fibroblast-related genes and genes identified in the WGCNA. These genes were related to cell proliferation and apoptosis and were involved in FoxO, Rap1, p53, Ras, MAPK, and PI3K-Akt pathways. Finally, a six fibroblast-related gene signature (*CCNB1*, *EGFR*, *E2F8*, *BTG1*, *TP63*, and *IGF1*) was identified and used for diagnostic model construction. The proportions of regulatory T cells and macrophages were significantly higher in keloid tissues than in controls.

Conclusion: The established model based on *CCNB1*, *EGFR*, *E2F8*, *BTG1*, *TP63*, and *IGF1* showed good performance and may be useful for keloid diagnosis.

Keywords: keloid; fibroblast; diagnostic model; immune cell

1. Introduction

A keloid is a fibroproliferative cutaneous disease following skin damage caused by a disruption in the balance between the proliferation as well as apoptosis of fibroblasts, resulting in excessive collagen deposition in the dermis and subcutaneous tissues [1]. Its clinical manifestations are scar hyperplasia and infiltrative growth outside the wound [2]. Keloid does not fade gradually and is often accompanied by itching as well as pain [2]. Importantly, uncontrolled keloids can contribute to functional impairment and a heavy burden on life [3]. Current treatments for keloids contain chemotherapy, surgery, radiotherapy, as well as stress therapy [4]. Although the treatment options can delay the progression of keloid, the recurrence rate is still high [5]. At present, the potential pathogenesis of keloid remains fully understood; therefore, in-depth investigations of the molecular mechanisms are necessary to promote the exploitation of novel treatment options and improve prognosis.

Multiple transcription factors, growth factors, as well as cytokines are reported to participate in wound healing [6]. Fibroblasts show differences in regulatory activity between normal wound healing and keloid tissues [7, 8]. During injury, dermal fibroblasts are activated to become myofibroblasts which can expressα-SMA, and then propagate and migrate into the wound, thus depositing the complex extracellular matrix (ECM) components, as well as forming a signaling niche [9, 10]. Once the integrity of the injury tissues is re-established, fibroblasts will become inactivated or begin to clear themselves [11]. On the contrary, if keloid fibroblasts always keep activated, and continuously secrete ECM, which can lead to excessive scar tissues [12]. Therefore, we can conclude that fibroblasts are the crucial effector cells in the formation of keloids; as well as targeting keloid fibroblasts may be a new therapy method for the treatment of keloids [13]. In addition to fibroblasts, genetic factors have pivotal functions in the occurrence of keloids [14]. Some genes related to fibrosis are associated with keloids [15, 16]. For instance, the TGF-β/SMADS signaling pathway has the close relation with the keloids. TGF-β is found to cause fibrosis in fibroblasts of keloids, participate in the SMAD signaling pathway, and enhance the expression of a variety of collagen genes as well as their corresponding protein productions [17]. Additionally, a series of genome-wide association studies have been employed to identify numerous genomic susceptibility loci for the occurrence of keloids. For example, neuronal precursor cell-expressed developmentally downregulated 4 (*NEDD4*) has been identified to be a candidate gene; it enhances the proliferation as well as the invasion of fibroblasts; while activates the transcriptional activity of TGF-β/catenin [18, 19]. However, genes related to fibroblasts in keloids have not been determined.

In the keloids, a chronic inflammatory state is common, in which multiple kinds of immune cells as well as cytokines are involved in the formation and development of keloids [20, 21]. Excavating the alterations of keloid immune microenvironment can

not only go deep into the pathogenesis of keloids, but also promote the treatment of keloid patients and improve the prognosis. As a result, this research screened a keloid fibroblast-related gene signature; as well as based on these genes, a diagnostic model was constructed for keloids. Furthermore, correlations between the identified signature genes and immune cell subsets were further explored. This study improves our understanding of the formation and progression of keloids and lays a foundation for diagnosis and treatment.

2. Data and methods

2.1. Expression profile data

On December 10, 2023, three datasets (GSE7890, GSE44270, as well as GSE212954) were downloaded from NCBI Gene Expression Omnibus (GEO) database. Among them, the GSE7890 dataset contained 19 relevant samples; 10 samples without drug treatment were selected, including 5 normal controls and 5 keloid regional tissue samples [22]. The detection platform for the GSE7890 dataset was the GPL570 Affymetrix Human Genome U133 Plus 2.0 Array. For the dataset of GSE44270, 12 samples were chosen, including 3 normal controls and 9 keloid region tissue samples, as well as its detection platform was the GPL6244 Affymetrix Human Gene 1.0 ST Array [23]. Additionally, the GSE212954 dataset contained 11 relevant samples, and 8 samples with clinical information were selected, including 4 normal controls and 4 keloid region tissue samples. The detection platform for the GSE212954 dataset was GPL20301Illumina HiSeq 4000. In total, 12 (5 + 3 + 4), and 18 (5 + 9 + 4) samples were incorporated in the control as well as keloid groups, respectively.

Because these datasets were derived from different batches of gene expression data, the sva package 3.38.0 in R 4.3.1 [24] was used to remove the batch effect on these three datasets; then, the combined expression data were obtained.

2.2. Evaluation of sample fibroblast scores

The gene set involved in fibrogenesis was downloaded from MSigDB in the Gene Set Enrichment Analysis (GSEA) database [25]. Then, the fibroblast scores for these samples were assessed through GSVA version 1.36.3 in R 4.3.1 [26]. Differences in the distribution of fibroblast scores between keloid as well as control samples were evaluated by the Kruskal–Wallis test. Additionally, the ability of fibroblast scores to identify sample types was analyzed using the receiver operator characteristic (ROC) curve method via pROC package version 1.12.1 in R 3.6.1 [27].

2.3. Weighted gene co-expression network analysis (WGCNA)

According to the all genes obtained in the combined dataset, WGCNA package version 1.61 [28] in R 4.3.1 was utilized to filtrate modules connected with the disease. The WGCNA algorithm was performed according to the steps of defining the adjacency function and module partition. The thresholds of module partitions were as follows: the

genes in the module set ≥ 200, as well as cutHeight = 0.995. After that, the correlations among each module, fibroblast score, as well as disease state were calculated, and the modules with significant correlations with both the disease state and fibroblast score were retained.

2.4. Analysis of differentially expressed genes

In the combined expression profile dataset, the DEGs between keloid and control samples were filtrated using the limma package version 3.34.7 in R 4.3.1 [29], with the thresholds of false discovery rate (FDR) < 0.05 and $|\log_2$ fold change (FC)$| > 0.5$. Afterwards, the identified DEGs were compared to the genes in modules obtained from WGCNA and fibroblast-related genes from GSEA database to acquire overlapping genes. Following, the overlapping DEGs were utilized for Gene Ontology (GO), as well as Kyoto Encyclopedia of Genes and Genomes (KEGG) pathway enrichment analyses using DAVID version 6.8 [30]. FDR < 0.05 indicated significance.

2.5. Construction of a diagnostic model on basis of the fibroblast-related DEGs

On account of the expression levels of the overlapping DEGs, rms version 6.3-0 [31] in R 4.3.1 was used for univariate logistic regression analyses, as well as genes with $P < 0.05$ were retained. These selected genes were evaluated with the LASSO algorithm using the lars package version 1.2 in R 4.3.1 [32].

The optimized fibroblast-related DEGs were extracted from the combined expression profile dataset (training dataset), and their expression levels were displayed. The support vector machine (SVM) method [33] was applied to construct a classifier for disease diagnosis according to these optimized DEGs (core: Sigmoid Kernel; cross: 10-fold cross validation), and the efficacy of the diagnostic model was evaluated using the ROC curve method in pROC package version 1.12.1 of R 3.6.1 [27]. In addition, the keloid-related expression profile (GSE185309) was downloaded as the validation dataset, and the expression levels of the corresponding fibrosis-related genes were extracted. Finally, the ROC curve method was also employed to verify model efficacy using the validation dataset (GSE185309).

2.6. Correlations with immune cell types

GSVA version 1.36.3 in R 4.3.1 was employed to evaluate the proportions of immune cell types in the combined expression profile dataset [26]. Differences in the proportion distribution of each immune cell type between the keloid as well as control groups were assessed by Kruskal–Wallis test implemented in R 4.3.1. Finally, the correlations between the differentially abundant immune cells, as well as the fibroblast-related DEGs used for model construction were evaluated based on Pearson correlation coefficients using the cor function in R 4.3.1.

2.7. Real-time quantitative PCR (RT-qPCR)

The expression of the optimized fibroblast-related DEGs (*CCNB1*, *EGFR*, *E2F8*, *BTG1*, *TP63*, and *IGF1*) was further validated in human tissues using RT-qPCR.

Samples from six patients with keloids were collected from the Plastic Surgery Hospital Chinese Academy of Medical Sciences (Beijing, China); as well as the keloid tissues and adjacent normal tissues were harvested. The study protocol was approved by the Ethics Committee of Plastic Surgery Hospital Chinese Academy of Medical Sciences, and informed consent was obtained from all participants.

Total RNA was isolated from the tissues using TRIzol reagent; as well as reverse-transcribed into cDNA using the PrimeScript 1st Strand cDNA Synthesis Kit (Takara, Kusatsu, Japan). The sequences of all primers were shown in Table 1, as well as *GAPDH* served as an internal control. The relative mRNA expression of the relevant genes were analyzed by the $2^{-\Delta\Delta CT}$ method. The data are reported as the mean ± standard deviation; as well as the Student's *t*-test in GraphPad Prism 5 was utilized for comparisons between two groups. $P < 0.05$ was considered significant.

3. Results

3.1. Screen of fibroblast-related genes, and assessment of sample fibroblast scores

A workflow of the bioinformatics analyses used in this study is shown in Figure 1. The samples in GSE7890, GSE44270, and GSE212954 before and after removing batch effects are shown in Figure 2A and 2B. Based on the GSEA database, seven biological processes in GO analyses related to fibroblast apoptosis and proliferation were involved, and 124 fibroblast-related genes were obtained (after removing duplicates). Then, the fibroblast score for each sample was evaluated using GSVA, revealing that the fibroblast scores were significantly higher in the keloid group ($P < 0.05$) than those in the control group (Figure 2C). Furthermore, the area under the curve (AUC) value in the ROC analysis was 0.926, suggesting the fibroblast score was a good way to identify the sample type (Figure 2D).

3.2. Selection of genes related to keloid and fibroblast scores by a WGCNA

The expression of all genes in the combined expression profile dataset were analyzed. In order to satisfy the premise of the scale-free network distribution as far as possible, the weight parameter power of the adjacency matrix was explored; network construction parameters were set and the scale-free distribution topology matrix was obtained. In Figure 3A, the value of power when the square value of correlation coefficient reached 0.9 for the first time was selected (i.e., power = 28); for this power value, the average node connectivity of the constructed co-expression network was 1, which fully conformed to the properties of the small-world network. Then, the dissimilarity coefficient between nodes was calculated, as well as the system clustering tree was obtained with the minimum number of genes in each module ≥200 as well as the pruning height set to cutHeight = 0.995. In total, 13 modules were obtained (Figure 3B). After that, the correlations among modules, fibroblast scores, as well as disease

status were calculated (Figure 3C). Among the 13 modules, five modules (blue, greenyellow, purple, tan, and turquoise) had significant correlations with both the disease state and fibroblast scores with absolute values of correlation coefficients ≥ 0.3; these modules contained 463, 222, 225, 213, and 637 genes, respectively. The genes related to the disease state and fibroblast score in these five modules were retained, including 1760 genes.

3.3. Identification of DEGs and functional analysis

Based on FDR < 0.05 as well as |$\log_2$ FC| > 0.5, 589 DEGs, including 440 down-regulated and 149 up-regulated genes, were identified in the keloid samples in comparison to the control samples (Figure 4A). In a comparison with genes obtained by the WGCNA (1760), and 124 fibroblast-related genes, 16 overlapping genes were found, including tumor protein p53-inducible nuclear protein 1 (*TP53INP1*), E2F transcription factor 8 (*E2F8*), cyclin A2 (*CCNA2*), Bcl2-associated X protein (*BAX*), cyclin B1 (*CCNB1*), B-cell translocation gene 1 (*BTG1*), *CKS2*, fibroblast growth factor 10 (*FGF10*), collagen type III alpha 1 (*COL3A1*), cyclin-dependent kinase regulatory subunit 1B (*CKS1B)*, insulin-like growth factor 1 (*IGF1*), superoxide dismutase 2 (*SOD2*), platelet-derived growth factor subunit B (*PDGFB*), secreted frizzled-related protein 1 (*SFRP1*), epidermal growth factor receptor (*EGFR*), and *TP63* (Figure 4B).

The 16 overlapping DEGs were further evaluated using GO and KEGG pathway analyses, revealing 19 significant GO terms in the biological process category and 14 KEGG pathways. According to the results of Figure 5A, these genes were involved in the GO terms "positive regulation of ERK1 and ERK2 cascade," "fibroblast proliferation," "cell division," "mitotic cell cycle phase transition," and "wound healing." Additionally, the overlapping DEGs were enriched in the following pathways: "Rap1 signaling pathway," "FoxO signaling pathway," "p53 signaling pathway," "Ras signaling pathway," "pathways in cancer," "MAPK signaling pathway," as well as "PI3K-Akt signaling pathway" (Figure 5B).

3.4. Construction and evaluation of a diagnostic model

On basis of the expression of the aforementioned 16 overlapping fibroblast-related DEGs, univariate logistic regression analyses were performed. Eight genes with $P <$ 0.05 were obtained, including *CCNB1*, *SERP1*, *EGFR*, *E2F8*, *TP63*, *BTG1*, *IGF1*, and *TP53INP1* (Figure 6A). Following a LASSO regression analysis, six optimized DEGs were identified, i.e., *CCNB1*, *EGFR*, *E2F8*, *BTG1*, *TP63*, and *IGF1* (Figure 6B).

The expression levels and efficiency of the identified six DEGs were analyzed in the combined training dataset and validation dataset. In the training dataset, the expression levels of *CCNB1*, *EGFR*, *E2F8*, *BTG1*, as well as *TP63* were remarkedly lower in the keloid samples than in the control samples ($P < 0.05$); however, the expression of *IGF1* was evidently enhanced in the keloid samples elative to the control samples (P <0.05, Figure 6C). Thereafter, a gene-based diagnostic model was

constructed, and the efficacy of the model was evaluated. Using the combined training dataset, the AUC values for the six genes were all above 0.7, whereas the combined AUC for the six genes was 0.958 (Figure 6D), indicating that the newly constructed diagnostic model based on the combination of the six genes had better diagnostic ability for keloids that those of each gene marker alone. Furthermore, the expression trends of the six genes in the different groups in the validation dataset were in keeping with those in the combined training dataset (Figure 6E). The AUC values for the six genes were all above 0.65, whereas the combined AUC for the six genes was 0.931 in the validation dataset (Figure 6F). The consistency in the results obtained using the combined training and validation datasets supported the reliability of the proposed diagnostic model.

3.5. Relationship between the optimized DEGs and immune cell types

After comparing the differences in the proportion distribution of immune cells between the keloid as well as control groups, two immune cell types were screened out, regulatory T cells and macrophages. Their proportions in the keloid groups were evidently risen ($P < 0.05$) in comparison with the control group (Figure 7A). In a correlation analysis of the optimized DEGs, fibroblast scores, and the two immune cells, *E2F8* was markedly negatively linked with the fibroblast score (cor. -0.474, $P < 0.01$); regulatory T cells had a significant negative correlation with *EGFR* (cor. -0.417, $P < 0.05$) as well as a significant positive correlation with *IGF1* (cor. 0.419, $P < 0.05$). In addition, macrophages had significantly positive correlation with *IGF1* (cor. 0.519, $P < 0.005$) as well as negative relationship with *EGFR* (cor. -0.583, $P < 0.005$), *TP63* (cor. -0.467, $P < 0.01$), as well as *BTG1* (cor. -0.399, $P < 0.05$) (Figure 7B).

3.6. Verification of the expression of the optimized fibroblast-related DEGs by RT-qPCR

The expression levels of *CCNB1*, *EGFR*, *E2F8*, *BTG1*, *TP63*, and *IGF1* were further validated in human tissues. In comparison to levels in control tissues, the expression levels of *CCNB1*, *EGFR*, *BTG1*, as well as *TP63* were evidently lower in the keloid tissues ($P < 0.05$), while the expression levels of *E2F8* as well as *IGF1* were evidently higher in the keloid tissues ($P < 0.05$, Figure 8). The outcomes exhibited the expression trends of *CCNB1*, *EGFR*, *BTG1*, *TP63*, and *IGF1* measured by RT-qPCR were in line with the results of the bioinformatics analysis (Figure 6C, E). The proportion of consistent results between RT-qPCR as well as bioinformatics analysis was 83.33%, indicating a relatively high reliability of our bioinformatics analysis.

4. Discussion

A keloids is a kind of benign fibrous tumor of the dermis, which poses a heavy psychological burden to patients [34]. Fibroblasts are key effector cells in the occurrence of keloids [13], as well as thus keloid fibroblast-related genes are potential therapeutic targets. In this study, 16 overlapping DEGs were identified; these genes were involved in various signaling pathways, containing the pathways of p53, MAPK,

and PI3K-Akt; as well as pathways in cancer. Finally, fibroblast-related gene signature including genes (*CCNB1*, *EGFR*, *E2F8*, *BTG1*, *TP63*, and *IGF1*) was used for diagnostic model construction. An ROC curve revealed that the model showed high sensitivity and specificity (based on AUC values). Additionally, regulatory T cells and macrophages showed higher relative frequencies in keloid tissues than in controls.

A recent study has shown that the growth characteristics of keloids may be attributed to an imbalance between rates of proliferation and apoptosis [35]. The MAPK signaling pathway is the classic pathway related to cell proliferation [36]. The p53 tumor suppressor has a central effect on cell apoptosis [37]. Similarly, PI3K-Akt signaling pathway is also related with the mediation of cell proliferation, differentiation, as well as apoptosis [38]. Of note, an imbalance between cell proliferation and apoptosis is also a feature of carcinogenesis. In this study, a pathway enrichment analysis revealed cancer-related pathways and microRNAs. In fact, keloids can exhibit various cancer-like features, for example, extremely high recurrence rates as well as uncontrolled progressive growth [39]. There is increasing evidence for interactions between pro-tumor factors and suppressors, which may explain its aggressive clinical behaviors. Moreover, the most striking similarities between keloids as well as cancer are their shared epithelial-mesenchymal transition, cellular bioenergetics, as well as epigenetic methylation signatures [40]. We speculated that these proliferation-, apoptosis-, and cancer-related pathways contribute to the progression of keloids. However, the specific mechanism needs to be further explored.

Based on the six fibroblast-related signature genes (*CCNB1*, *EGFR*, *E2F8*, *BTG1*, *TP63*, and *IGF1*), a diagnostic model was developed in this study. *CCNB1* was enriched in cell cycle-related functions and the p53 signaling pathway. Keloids are featured by dermal germination beyond the original margin of the wound, depending on the ectopic cell cycle [34]. For p53, compaed to the normal scar tissues, it expression was often higher [41]. Importantly, Wang et al. [42] have also reported that changes in *CCNB1* may be involved in keloid formation by regulating cell cycle and p53 signaling pathway. Huang et al. [43] demonstrated *CCNB1* expression was decreased in keloid fibroblasts treated by 5-fluorouracil, suggesting that CCNB1 may serve as a treatment target for keloids. In addition to *CCNB1*, *IGF1* is involved in the p53 signaling pathway, and may play functions in the pathogenesis of keloids. EGFR can activate the tyrosine protein kinase-binding domain of the receptor when bound to its corresponding ligand EGF, which further activates downstream signaling pathways, thereby causing cell division and proliferation [44]. It has been reported that EGFR is down-regulated in keloid tissues [45], consistent with our results. EGF-induced fibroblasts in keloid tissues show lower mobility than those of fibroblasts in normal tissues and undergo reduced mitosis [46]. Therefore, the down-regulation of EGFR may promote the development of keloids. Both *E2F8* and *BTG1* were associated with cell proliferation. *TP63* and *IGF1* were also

involved in some cancer-related pathways. To our best knowledge, the effects of these four genes on keloids have not been investigated. These fibroblast-related genes may contribute to the cancer-like features of keloids. ROC curves revealed that the AUC values of the six genes were all above 0.7, whereas the AUC value for the combination of the six genes was 0.958, supporting the high predictive value of the model [47, 48]. Taken together, we can infer that these six genes are candidate genes in keloids, and the model based on these six genes can be used for diagnosis. However, the exact roles of these genes need to be further studied.

In addition, infiltration of immune cells is a hallmark of keloid tissues; as well as preferential collection of immune cells regulates skin repair process through interactions with keloid fibroblasts [49]. There is increasing recognition of the abnormal immune cell composition as well as activity in nonlesional skin of patients with keloids [50, 51]. Our research observed regulatory T cells and macrophages have higher relative frequencies in keloid tissues than in controls. Studies have illustrated the number of regulatory T cells is increased in the lesions of keloid skin [52, 53]. These cells promote the preferential accumulation of type III collagen in the presence of anti-CD3/CD28 [52]. Importantly, the accumulation of regulatory T cells at sites of injury can regulate the polarization of M1 to M2 macrophages [54]. The feature of the common wound healing process is an orderly interim from a M1 macrophages-dominated inflammatory phase at early stage to a M2 macrophages-dominated recovery phase [55]. Disruption of this progression can contribute to the prolonged inflammation, delayed wound healing or increased scarring. The proportion of M2 macrophages is abnormally elevated in keloid lesions [56, 57], consistent with our findings. Specially, the two kinds of immune cells were significantly negatively linked to *EGFR* and *TP63*; as well as positively associated with *IGF1*. Nevertheless, detailed relationships between different types of immune cells as well as fibroblast-related genes are still necessary to beinvestiagted.

This study had some limitations. For example, the samples size was small, and further experiments using a larger sample size are needed. Additionally, our study was based on bioinformatics analyses; in vitro and in vivo experiments are warranted to further explore specific roles and mechanisms of actions of the six optimized DEGs and two immune cell types in keloid development.

Conclusion

A fibroblast-related diagnostic model for keloid based on a six-gene signature (*CCNB1*, *EGFR*, *E2F8*, *BTG1*, *TP63*, and *IGF1*) was proposed, showing high predictive accuracy in keloid diagnosis. Furthermore, our results supported the critical roles of regulatory T cells and macrophages in keloid formation. All the findings provide a basis for the early diagnosis as well as control of keloids, thereby improving

clinical outcomes.

797601503589171200_MaGuiE_Comprehensive bioinformatic analysis reveals a fibroblast-related gene signature for the diagnosis of keloids.docx

ORIGINALITY REPORT

19%	16%	16%	1%
SIMILARITY INDEX	INTERNET SOURCES	PUBLICATIONS	STUDENT PAPERS

PRIMARY SOURCES

1	www.ncbi.nlm.nih.gov<br>Internet Source	3%
2	www.frontiersin.org<br>Internet Source	2%
3	peerj.com<br>Internet Source	1%
4	文成 赵, lin wang, Xiangping XU. "Identification of Important Genes of childhood autism and Construction of the Diagnostic Model", Research Square Platform LLC, 2024<br>Publication	1%
5	www.science.gov<br>Internet Source	1%
6	Jiaheng Xie, Liang Chen, Yuan Cao, Dan Wu, Wenwen Xiong, Kai Zhang, Jingping Shi, Ming Wang. "Single-Cell Sequencing Analysis and Weighted Co-Expression Network Analysis	1%

Based on Public Databases Identified That TNC Is a Novel Biomarker for Keloid", Frontiers in Immunology, 2021
Publication

7	Zhankui Jin, Fuqiang Liu, Guoan Zhang, Jingtao Zhang, Xiangrong Zhao, Xueping Huo, Xiaoyan Huang, Cuixiang Xu. "An effective disease diagnostic model related to pyroptosis in ischemic cardiomyopathy", Journal of Cellular and Molecular Medicine, 2023<br>Publication	1%
8	bmcoralhealth.biomedcentral.com<br>Internet Source	1%
9	www.hindawi.com<br>Internet Source	1%
10	www.researchsquare.com<br>Internet Source	<1%
11	Yan Wang, Tao Liu, Yan Liu, Jun Chen, Benqiang Xin, Maoyuan Wu, Weigang Cui. "Coronary artery disease associated specific modules and feature genes revealed by integrative methods of WGCNA, MetaDE and machine learning", Gene, 2019<br>Publication	<1%
12	Li Sha, Zhangxiang Wu, Zichen Ling, Xingxin Liu, Xiaoyan Yu, Shuting Zhang.	<1%

"Dewaterability and energy consumption of electro-dewatered sludge near the anode and the cathode during electro-dewatering process", Journal of Environmental Chemical Engineering, 2021

Publication

---

13 Zhe Jin, Ya-Hui Liu. "Metabolic–related gene signatures for survival prediction and immune cell subtypes associated with prognosis in intrahepatic cholangiocarcinoma", Cancer Genetics, 2023 <1%

Publication

---

14 www.besjournal.com <1%

Internet Source

---

15 www.mdpi.com <1%

Internet Source

---

16 Haili Jin, Wei Liu, Weiming Xu, Liping Zhou, Huarong Luo, Cheng Xu, Xi Chen, Wenbin Chen. "Identification of Prognostic Factors in Cholangiocarcinoma Based on Integrated ceRNA Network Analysis", Computational and Mathematical Methods in Medicine, 2022 <1%

Publication

---

17 Submitted to Queen's University of Belfast <1%

Student Paper

---

18 www.omicsdi.org <1%

Internet Source

19	iopscience.iop.org<br>Internet Source	<1 %
20	Qinqing Li, Hongfeng Dai, Fengming Ran, Yueyuan Luo, Jingyan Gao, Ailin Deng, Nan Xu, Chengde Liao, Jun Yang. "Cranial irradiation-induced impairment of axonal transport and sexual function in male rats and imaging of the olfactory pathway by MRI", NeuroToxicology, 2022<br>Publication	<1 %
21	pubmed.ncbi.nlm.nih.gov<br>Internet Source	<1 %
22	www.brazilianjournals.com<br>Internet Source	<1 %
23	A. Salami, J. Eriksson, L. Nyberg. "Opposing Effects of Aging on Large-Scale Brain Systems for Memory Encoding and Cognitive Control", Journal of Neuroscience, 2012<br>Publication	<1 %
24	Yuntian Zhang, Tzong-Yi Lee. "Revealing the Immune Heterogeneity between Systemic Lupus Erythematosus and Rheumatoid Arthritis Based on Multi-Omics Data Analysis", International Journal of Molecular Sciences, 2022<br>Publication	<1 %
	ir.lib.uwo.ca	

25	Internet Source	<1%
26	Chunxiao Wu, Qiquan Yu, Weizhen Shou, Kun Zhang, Yang Li, Wentao Guo, Qi Bao. "Co-stimulatory molecule clusters correlate with survival, immune infiltration, and tumor mutation burden in non-small cell lung cancer", All Life, 2022<br>Publication	<1%
27	mdpi-res.com<br>Internet Source	<1%
28	iv.iiarjournals.org<br>Internet Source	<1%
29	www.dovepress.com<br>Internet Source	<1%
30	Bo Chen, Chun Lin, Xing Jin, Xibin Zhang, Kang Yang, Jianjian Wang, Feng Zhang, Yuxin Zhang, Yingying Ji, Zhaoxiang Meng. "Construction of a diagnostic model for osteoarthritis based on transcriptomic immune-related genes", Heliyon, 2023<br>Publication	<1%
31	Jingxian Li, Zheng Kong, Yuanjiong Qi, Wei Wang, Qiang Su, Wei Huang, Zhihong Zhang, Shuai Li, E Du. "Single-cell and bulk RNA-sequence identified fibroblasts signature and CD8+ T-cell - fibroblast subtype predicting	<1%

prognosis and immune therapeutic response of bladder cancer, based on machine-learning bioinformatics retrospective study", International Journal of Surgery, 2024
Publication

32	assets.researchsquare.com<br>Internet Source	<1%
33	tessera.spandidos-publications.com<br>Internet Source	<1%
34	www.spandidos-publications.com<br>Internet Source	<1%
35	Elenbaas, Jared S.. "Characterizing the Causal Role of SVEP1 in Human Disease", Washington University in St. Louis, 2024<br>Publication	<1%
36	Tingjun Liu, Ankang Hu, Quangang Chen, lianlian Wu, Lingzhi Zhang, Dandan Qiao, Zhutao Huang, Tianyuan Lu, Jie Wang. "The prognostic value of bioinformatics analysis of ECM receptor signaling pathways and LAMB1 identification as a promising prognostic biomarker of lung adenocarcinoma", Research Square Platform LLC, 2023<br>Publication	<1%
37	Xiaocheng Guo, Xinyuan Feng, Yue Yang, Wenying An, Lunhao Bai. "Machine learning-based identification and immune	<1%

characterization of ferroptosis-related molecular clusters in osteoarthritis and validation", Aging, 2024
Publication

Exclude quotes Off
Exclude bibliography On
Exclude matches Off
